# American Medical Association

**Published:** 1848-06

**Authors:** 


### THE WESTERN JOURNAL.

THIRD SERIES.—VOL. I.—NO VI.

LOUISVILLE, JUNE 1, 1848.

### AMERICAN MEDICAL ASSOCIATION.

On the first Tuesday in May, according to previous announcement, the American Medical Association met in Baltimore. The number of Delegates in attendance was two hundred and seventy-seven, comprising representatives from about twenty States, and most of the Medical Schools and Societies in the country. The officers of the Association present were—Dr. Chapman, of Philadelphia, President; Dr. Knight, of New Haven, and Dr. Stevens, of New York, Vice Presidents; Dr. Stillé, of Philadelphia, and Dr. Dunbar, of Baltimore, Secretaries; and Dr. Hays, of Philadelphia, Treasurer. Two of the Vice Presidents, Dr. Moultrie, of Charleston, and Dr. Buchanan, of Nashville, were absent. The western and south-western States were not fully represented; only about fifteen delegates were present from all the States west of the mountains, and these were from Ohio, Indiana, Illinois, Missouri, Tennessee, and Kentucky. It is not, however, to be inferred from this that our brethren in those quarters are indifferent to the objects of the Association. The reverse, we are persuaded, is the fact, of which they will give full proof when the Association shall meet, as it will do at an early day, in one of our western cities.

The opening address of the President disappointed us, as we believe it did most of the members. We expected something studied and elaborate—a discourse carefully prepared, setting forth the aim and tendencies of the Association, or indicating the progress of medical science during the past year. Professor Chapman favored the Association with nothing of the sort, but addressed the body in a few extemporaneous remarks pronounced in the handsomest manner, but hardly doing justice to the profession, which he represented as having ceased to command the homage of society which it once enjoyed—as having, in some degree, lost caste, and become "tainted."

We are sure that the late venerable President of the American Medical Association is the last man who would afford "aid and comfort" to the enemies of his profession, but it cannot fail to strike any one that such representations are calculated to give peculiar pleasure to those who are in the habit of decrying scientific medicine. The error no doubt grew out of the unpremeditated character of the remarks, for no one is more fully persuaded than Professor Chapman that our profession was never more progressive or more respectable than it is at the present day, and that it never embraced so large a number of truly scientific and enlightened practitioners. It was enough to be present at the Association and listen to the reports and remarks of its members, to be satisfied that this is not more true of the profession in any country than our own; and we confess that to us it was one of the gratifying circumstances of the meeting that, while much was said about improvement, very few speakers used the word "reform." It was evident, that the idea the minds of the vast majority of the members was, not to *reform* the profession, but to carry it forward in its career of advancement; and such is the tendency of the annual meetings of the Association, from which will go forth influences that cannot fail to be felt for good throughout the land.

The election of officers, which followed soon after the delivery of the address by the President, excited much interest, and occupied an unnecessary amount of time. The committee of nomination were unwisely instructed to report to the Association three names for each office, to which members were allowed subsequently to add as many more as they pleased. The result was, that in the scattered vote only three officers were elected on the first ballot—Professor Jackson, Vice President, Dr. Stillé, Secretary, and Dr. Hays, Treasurer, all of Philadelphia. A second ballot consumed the afternoon session, and before the result was stated by the tellers, next morning, Dr. Hays offered a resolution to the effect that, inasmuch as many members had voted on the second ballot under a misapprehension of certain restrictions placed upon the nominations, it be set aside, and a committee appointed to *report* officers to the Association. This resolution was adopted, and the ballot accordingly set aside. Dr. Jackson then rose and after stating that he thought it would conduce to the harmony of the meeting to let the committee about to be appointed report *all* the officers, resigned the office which had been conferred upon him the evening before. Under the same view of the case, Dr. Hays and Dr. Stillé also resigned.

In the discharge of their duty the nominating committee recognised the claims of the several great divisions of our country, as will be seen by the following list of officers presented by them to the Association:

Dr. Alexander H. Stevens, of New York, President.

Dr. John C. Warren, of Boston, Dr. Samuel Jackson, of Philadelphia, Dr. Paul F. Eve, of Augusta, Georgia, and Dr. W. M. Awl, of Columbus, Ohio, Vice Presidents.

Dr. Alfred Stillé, of Philadelphia, and Dr. H. J. Bowditch, of Boston, Secretaries.

Dr. Isaac Hays, of Philadelphia, Treasurer.

These nominations were unanimously concurred in by the Association, and thus the matter which had already taken up too much time was summarily disposed of. The President elect was conducted to the chair, who upon taking his seat acknowledged the honor thus conferred upon him in a few appropriate remarks.

A report from the Treasurer was then received in reference to the assessment necessary for meeting the expenses of the ensuing year. According to a provision of the constitution the annual assessment cannot exceed three dollars. The transactions of the Association, he conjectured, would make a volume of eight hundred pages, and the expenses for the year would probably amount to sixteen hundred dollars. The deficit, whatever it might be, he thought, might be made up by voluntary contributions from Institutions and Societies, and by the sale of the transactions.

The annual report of the standing committee on Obstetrics was then read by the chairman of the committee, Dr. Hervey Lindsley, of Washington City. The report was chiefly devoted to the history of Etherization in obstetrical practice, and presented a highly favorable view of the new agents. Dr. Lindsley is a decided a lvocate for the use of anæsthetics in certain cases of parturition. It is hardly necessary to say that not a few members dissented from his views, and that in regard to the whole subject of etherization there was much diversity of opinion in the Association.

A communication from the College of Pharmacy, of New York, relative to the importation of spurious drugs into this country, was received and read. Dr. Edwards, a member of Congress from Ohio, being present, read to the Association a highly interesting document on the same subject. He entered into details, showing that the evil had grown to be an enormous one. The articles of the materia medica in general use are now so frequently adultered, that the physician cannot be too careful in the selection of his medicines. Quinine, opium, scammony, iodine, and hydriodate of potash are among the articles most liable to adulteration, and it would appear that they are sometimes adulterated, "to order," for the Western market. It is high time that this matter was looked into by our government. Dr. Edwards read a law which he had drawn up providing for the appointment of competent persons as examiners of all imported drugs at the different seaports, and urged upon the Association the necessity of immediately memorializing Congress in favor of its enactment. He had strong hopes that, by the concerted action of the profession, he would be able to get it through Congress at its present session.

The report of the standing committee on Surgery was then read by the chairman of the committee, Dr. George W. Norris, of Philadelphia, and was listened to with marked attention. It presented a clear and able narrative of the progress of Surgery during the past year, and embraced the history of some important surgical cases. It will form a part of the volume of transactions which the profession may soon expect, and when it appears we hesitate not to promise that it will be deemed a valuable contribution to our professional literature.

Dr. Isaac Parrish, of Philadelphia, a member of the same committee, also read a report relative to the use of anæsthetics in Surgery. An admirable summary of what had been effected by these agents in different countries was given, and an opinion was expressed by the writer favorable to their employment.

The reading of Dr. Parrish's report occupied the Association up to the hour of taking recess. In the afternoon, the report on American Medical Literature was read by Dr. Oliver Wendell Holmes, of Boston, chairman of the committee. The reputation of the author for wit and poetry, as well as for professional learning and critical acumen, had excited in the members of the Association great curiosity to hear this report. We did not hear the conclusion of the report and are not able, therefore, to speak of it as a whole, but so much of it as we heard appeared to us just, able and judicious. Some of the strictures of the writer were severe, but, we thought, not unmerited. Many of the members thought that he evinced a disposition to sneer at our medical literature, and was partial in his retrospect and comments. He was charged with showing an overweening regard for the Journals published near Boston, and overlooking the merits of others more remote. But it is easy to perceive how, from the very nature of his task, it was impossible for Dr. Holmes to satisfy all his hearers. The Association was made up, in large measure, of men who had aided in swelling the volume of American medical literature. In a rapid survey, such as he was obliged to take in a report like this, of so wide a field, it ought not to surprise any one, that he failed to do justice to many; and if he was attracted longer by familiar names than by others, perhaps equally meritorious, of which, in the distance, he had heard less, this is precisely what must occur to every one who writes on such a subject. We shall be disappointed if Dr. H.'s report is not deemed a very able one.

After the reading of Dr. Holmes' report, the subject of etherization was discussed until the hour arrived for adjourning. Professor Warren of Boston, presented his views at length. Dr. W. is known to be one of the warmest advocates in the country for etherization. He reported a number of cases in which he had lately seen the best effects from the practice; but he is disposed to regard chloric ether as a preferable agent to either sulphuric ether or chloroform. Time did not afford other members an opportunity to express their views, some of which would have been widely variant from those of Professor Warren. We shall recur to this subject again.

Thursday morning, the standing committees were appointed, a list of which and of the members composing them will be given hereafter. The Association then proceeded to determine on the next place of meeting, and this question gave rise to some debate. Some members were fearful, that, if the Association continued to hold its meetings in the Atlantic cities, the West would become dissatified and start a separate organization. On the other hand it was contended, that at present the west was hardly prepared to do herself justice, since she had comparatively but few medical societies, and could not send many delegates to the Association, even if it should meet in one of her own cities, but that another year being given, associations would be formed in all parts of the country, and a full representation be ready to welcome it to the west. It was decided, in the end, to meet next spring in Boston, with the understanding that the year after the place of meeting should be Cincinnati.

Dr. Wellford, of Fredericksburg, Va., now proceeded to read the report of the committee on Medical Education, of which Dr. Stevens was chairman. The report was accompanied by a number of resolutions, three of which had an important bearing upon medical schools, and in substance are as follows: 1st. That the plan of education in any medical school must be considered radically defective, which does not embrace anatomical dissections by the pupils, and clinical instruction. 2d. That it be recommended to medical schools in the country which have not already done so, to extend their sessions to five months. 3d. That it be recommended to the Faculties of the several medical schools to associate with them, in the examination of their students for the doctorate, a number of physicians not connected with the schools.

The first of these resolutions passed unanimously. The second was adopted with little division, although it is known that not a few gentlemen in the Association connected with medical schools, question the policy of lengthening the lecture term. Upon the third, the vote was by no means unanimous. No topic came before the Association concerning which the sentiments of the members were so discordant as that of medical education, and it was but too apparent that they were most confident in their schemes of innovation who had enjoyed least experience as teachers. But, upon the whole, although there were radical and extravagant suggestions made by a few, great moderation and freedom from narrow prejudices characterized the decisions of the Association on this important subject. As to the third resolution, under the present arrangement in most of the schools, we suppose it will prove a nullity. Physicians qualified to examine students will not be found willing to undertake, gratuitously, the responsible task of deciding upon the qualifications of candidates for the degree of doctor of medicine, and, Professors regard the fee paid by graduates as hardly an adequate remuneration for their own services.

Professor Smith, of New York, chairman of the committee on Practical Medicine, reported that he had unfortunately lost his baggage on his way to Baltimore, and with it his report. He stated briefly the leading points embraced in his report, which document he was requested to furnish to the committee on publications. His friends expected from him an able paper, and we shall look with interest for it among the proceedings of the Association.

Professor Dickson, of New York, was not present, but it was announced that his report on the Medical Sciences was at the disposal of the Association. It was referred to the Committee on publications, as were a number of other interesting communications, among which was a paper by Dr. Buck, of New York, describing a fascia of the penis lately discovered by him.

A communication from the National Institute on the subject of Hygiene was read by Dr. Winne, of Baltimore, which led to the appointment of a committee to investigate the sources of disease in the principal cities and towns in the United States. Valuable statistics will, no doubt, be laid before the Association at its next meeting touching the subject of Hygiene.

Dr. Atlee offered a resolution recommending the formation of State Medical Societies, where none now exist, which was adopted. We hope the suggestion will meet with a hearty response from the profession, and that no State will long be without a medical society.

An incident occurred on the first day of the meeting to which it is proper that we should allude, more particularly as it has been made the subject of remark in some of the religious newspapers, and if not explained may lead to impressions unfavorable to the religious character of the Association. When the committee for the nomination of officers had retired, Dr. Grafton Tyler, of Washington city, offered a resolution that the daily sessions of the Association be hereafter opened with prayer, and instructing the committee of arrangements to procure the services of such ministers as they saw fit. This resolution ought to have prevailed without a word of comment or opposition, but, constituted as the association was, such a result could hardly have been expected, and therefore it was unfortunate that such a proposition was submitted. Among men of every religious creed, accustomed to express their sentiments with the utmost freedom, it might have been foreseen that there were those who would object to such a procedure. Dr. Thomas, of Baltimore, belonging to the society of Friends, opposed it, and he was sustained by Dr. Bond of the same city, though upon different grounds. The service, he said, would be agreeable to him, and probably to the many; but since there were those to whom it would be unpleasant, he hoped Dr. Tyler would withdraw his resolution. Dr. T. replied that he had offered it under a sense of duty and could not withdraw it. Dr. Stewart, of New York, moved to lay it on the table, which after some discussion was withdrawn. The motion to lay on the table was immediately renewed by half a dozen members, and upon the question being taken it was determined in the affirmative.

In the afternoon the subject was revived by a resolution, offered by Dr. Reese, of New York, to the effect that the Association desired it to be understood that in laying the resolution, in relation to opening the sessions with prayer, on the table, they disclaim any want of respect for devotional exercises on such occasions, and that the action must be regarded in the light of a concession to those members who had scruples of conscience on the subject. The discussion which ensued upon this resolution was the warmest elicited at the Association. Dr. Harris, of Tennessee, spoke with great earnestness and feeling in favor of the original proposition. When he concluded, Dr. Kreider, of Ohio, moved to amend the resolution by substituting that a certain portion of Scriptures should be read by the President every morning. He was followed by Professor Harrison, of Cincinnati, in a most fervid speech, in the course of which he was so unfortunate as to refer to our age and country as "protestant." His language brought on a tempest of cries for "*order*," and "*question*," during which he sat down. The resolution of Dr. Reese was adopted.

The resolution expressed the sentiments of the Association. It was felt to be inexpedient to press the first proposition, precisely as in the Board of the American Bible Society, composed of Christians of various denominations, it was found necessary, in the beginning, to adopt a law that their meetings for business should not be opened with prayer. A portion of Scripture is read, but prayers are excluded, because a form could not be agreed upon. If the American Bible Society has experienced the necessity for such a rule, certainly the American Medical Association, embracing Jews and Christians, Catholics and Protestants, may be excused for having yielded to its necessity. We cannot doubt that this explanation will be satisfactory to our friends who have felt aggrieved by the decision of the Association.

We have not reported as fully as we intended the doings of the Association, but find ourselves arrived at the limits assigned us, and must therefore reserve for our next number the remainder of our narrative.

The meeting to us was one full of interest, and inspired us with higher hopes for our profession. We trust that the Association may now be considered firmly established, and that its annual meetings will be invested with a continually increasing interest.

After a most harmonious session of four days, the Association adjourned on Friday, the 5th of May, at 11 o'clock, A.M., to meet in Boston on the first Tuesday in May, 1849.

